# Research on Temperature Distribution Reconstruction of Deflagration Fields via Spectral-Image Fusion

**DOI:** 10.3390/s26123746

**Published:** 2026-06-12

**Authors:** Meng Zhao, Maoyong Bai, Zhaojun Wu, Shaodong Bai, Zheng Qiu, Kang Du, Yong Tan, Hongxing Cai

**Affiliations:** Key Laboratory of Spectral Detection Science and Technology, School of Physics, Changchun University of Science and Technology, Changchun 130022, China; zhaomeng@cust.edu.cn (M.Z.); baimaoyong@mails.cust.edu.cn (M.B.); baishaodong@mails.cust.edu.cn (S.B.); qiuzheng1937@163.com (Z.Q.);

**Keywords:** deflagration temperature field, spectral thermometry, non-contact thermometry, least squares method, spectral image fusion

## Abstract

Multispectral temperature measurement technology based on blackbody radiation theory has been widely applied in the field of non-contact temperature measurement. However, its applicability is limited by the single-point measurement mode. To address this limitation, this study developed a spectral fusion temperature measurement device and proposed a new method for reconstructing the two-dimensional temperature field of deflagration fireballs by fusing spectral and imaging data. The device adopts a CCD sensor and a fiber optic spectrometer placed in parallel with parallel optical axes. To ensure the accuracy of the CCD’s response characteristics at different distances, the photo-response non-uniformity (PRNU) calculation method was used for precision validation. In this study, spectral and imaging data of deflagration fireballs were obtained through experiments. Spectral data of consecutive frames at 189 ms, 192 ms, 195 ms, and 198 ms were extracted and analyzed, confirming that the temperature range at the four time points is 1050 K to 1800 K. The proposed method generates temperature elements with equal temperature intervals and their probabilities within the temperature range, and calculates the theoretical radiation spectrum of each element. Then, least squares optimization fitting is performed on the experimentally measured spectra to obtain the optimal probabilities of the temperature elements in the temperature field. By combining these optimal probabilities with CCD grayscale images, the 2D temperature distribution of the deflagration fireball was reconstructed. Results show that: the PRNU value of the device at a distance of 9 m is less than 2.2% through experimental verification; fused images of the temperature field spectra of four consecutive frames of the deflagration fireball were obtained using the proposed method. The average temperatures reconstructed by the proposed method at 189 ms, 192 ms, 195 ms, and 198 ms were 1382 K, 1373 K, 1366 K, and 1357 K, respectively, while the corresponding temperatures obtained by conventional spectral inversion were 1430 K, 1422 K, 1414 K, and 1406 K. The relative errors were 3.2%, 3.4%, 3.3%, and 3.4%, respectively, with an average relative error of approximately 3.3%.

## 1. Introduction

Temperature is a critical parameter for evaluating damage effects in deflagration temperature fields, as it reflects the thermal damage efficacy and energy transfer dynamics of weapons [1]. The explosion temperature field serves as a key indicator for assessing the destructive performance of fuel-air explosives, thermobaric explosives, and cloud detonation weapons [2]. Investigating temperature variations during explosions is essential for the research, development, standardization, and production of weapon systems [3]. With advancements in thermobaric explosive technology, diverse testing methods for deflagration fireballs and studies on thermal damage effects have garnered significant attention [4]. Currently, theoretical calculations and numerical simulations cannot accurately determine temperatures in explosion fields, necessitating experimental measurements [5]. The transient nature of explosion temperature fields, characterized by short duration, wide temperature fluctuations, and extreme peak values, poses substantial technical challenges. Conventional thermometric methods often fail to meet these demands, making the measurement of explosion temperature fields a prominent yet challenging research focus in recent years.

Spectroscopic thermometry, characterized by its non-contact operation and rapid response, is particularly suitable for high-temperature transient deflagration fields. For example, Wang et al. measured fireball temperatures in transient high-temperature environments using multi-wavelength spectroscopy [6]. Fourier transform, Savitzky–Golay smoothing, spectral data normalization, and least-squares fitting were used to process and correct the spectral data. The corrected solar spectral curve was then used to calibrate spectral data obtained from different spectrometers, demonstrating the applicability of this approach in complex deflagration scenarios. Guo et al. proposed a method for measuring the plume temperature of solid rocket engines based on flame radiation spectroscopy [7]. A 350–1000 nm fiber-optic spectrometer was used to construct a measurement system for the flame radiation spectrum of the engine plume. The spectral response coefficients were calibrated using a standard radiation blackbody furnace, yielding a curve of response coefficients as a function of wavelength. This curve was subsequently applied to correct the measured radiation spectrum data of the plume. It was verified that the flame radiation of the engine plume in the 675–745 nm band can be approximated as graybody radiation.

In addition to spectroscopic methods, imaging and reconstruction techniques have also been applied to high-temperature fireball diagnostics. Liu Xiaowei et al. integrated CCD imaging technology with multi-criteria iterative reconstruction algorithms to propose a method for three-dimensional temperature field measurement of high-temperature fireballs, laying the groundwork for high-precision thermometry in such fields [8]. To address challenges in explosion fields, such as the high temperature, large temperature dynamic range, inability to measure directly, and difficulty in achieving high precision in temperature field reconstruction, an improved simultaneous algebraic reconstruction technique (SART), widely used in image reconstruction, was proposed to realize temperature field reconstruction in explosion environments. This improved method replaces the fixed iteration step size of the original algorithm with an adaptive step size. The iteration step size is determined by assessing whether distortion occurs between adjacent pixels: a larger step size is used when no distortion is detected, while a penalty is applied to reduce the step size when distortion occurs. Furthermore, a uniformity criterion is incorporated into the least-squares criterion satisfied by the SART algorithm to enhance reconstruction quality.

Image-based pyrometry and camera calibration methods have also been widely investigated for flame and high-temperature object temperature measurements. Svensson et al. calibrated an RGB CCD camera and interpreted two-color images for soot KL factor and flame temperature [9]. Their method uses the published spectral response of the CCD combined with monochromator-based calibration, but the retrieved temperature represents a line-of-sight path-integrated value weighted by soot distribution, making it difficult to resolve spatially non-uniform temperature fields. Sala et al. proposed a camera calibration method based on quantum efficiency estimation from blackbody images, followed by a neural network for pixel-wise temperature prediction [10]. While this approach extends the measurable temperature range (700–3500 °C), the reported accuracy is on the order of tens of degrees, and the reliance on synthetic training data limits its robustness when applied to transient flame spectra with unknown emissivity characteristics. Toro et al. combined a color imaging camera with spectral reconstruction and two-color pyrometry to estimate flame temperature from reconstructed spectra [11]. However, spectral reconstruction from only three broadband channels inevitably loses spectral detail, and the method remains fundamentally a two-color approach, which is sensitive to camera calibration accuracy, emissivity assumptions, and soot concentration variations. Collectively, these imaging-based methods provide valuable spatial temperature-related information and excel in visualization and temporal resolution, but they are typically sensitive to detector response calibration, optical path effects, emissivity assumptions, and background radiation. Moreover, they face fundamental difficulties in providing reliable quantitative temperature reconstruction when the radiation field is strongly non-uniform or when spectral characteristics vary rapidly during transient deflagration.

Despite the progress made by these studies, existing methods remain insufficient for deflagration fireball thermometry, which demands simultaneous satisfaction of non-contact operation, high temporal resolution, quantitative temperature retrieval, and two-dimensional spatial reconstruction. Contact methods (e.g., thermocouples) are fragile under extreme shock and disrupt the original field. Spectroscopic methods provide rapid, non-contact line-of-sight temperature but lack spatial resolution. Imaging methods capture transient morphology but cannot directly convert grayscale to temperature without intricate calibration. Tomographic approaches require multi-view access and complex geometric calibration that is impractical in transient explosion tests.

To address these challenges, this study proposes a spectral-image fusion method that couples spectral unmixing with CCD-based spatial mapping to reconstruct the two-dimensional temperature distribution of deflagration fireballs. Unlike the methods reviewed above, which either provide single-point or path-integrated temperature (Refs. [6,7,8]), rely on color-ratio-based inference from limited spectral bands (Refs. [9,11]), or employ neural-network-based regression requiring extensive calibration data (Ref. [10]), the proposed approach uses directly measured spectra to retrieve multiple temperature elements with physically interpretable probability weights via nonlinear least-squares spectral unmixing. These weights are then coupled with CCD grayscale images, whose response non-uniformity is calibrated using the photo-response non-uniformity (PRNU) method, to achieve pixel-resolved temperature reconstruction. In this way, the proposed method extends spectral thermometry from single-point temperature inversion to quantitative two-dimensional temperature field reconstruction, combining the spectral accuracy of direct spectroscopy with the spatial mapping capability of imaging. The key contributions are: (1) a spectral-image fusion framework with a coaxial fiber-optic spectrometer and imaging sensor; (2) a nonlinear least-squares spectral unmixing algorithm that decomposes the measured spectrum into weighted temperature elements; and (3) PRNU-based imaging sensor calibration to improve the reliability of spatial temperature mapping.

The advantages of the proposed method over existing imaging-based pyrometry approaches can be summarized as follows. First, unlike Svensson et al. [9], whose two-color method yields path-integrated temperatures weighted by soot distribution and cannot resolve spatial non-uniformity, the proposed spectral unmixing retrieves multiple temperature elements with independent probability weights, enabling the reconstruction of a two-dimensional temperature field that captures the internal structure of the fireball. Second, in contrast to Sala et al. [10], who rely on a neural network trained on synthetic blackbody data with reported accuracy on the order of tens of degrees, the proposed method employs a physics-based nonlinear least-squares fitting framework that is directly grounded in Planck’s law and does not require extensive training data. This physical interpretability makes it inherently more robust when applied to transient deflagration spectra with unknown emissivity characteristics. Third, whereas Toro et al. [11] reconstruct flame spectra from only three broadband color channels—a process that inevitably discards spectral information—the proposed method directly measures the full fiber-optic spectrum (350–1000 nm), preserving complete spectral detail for temperature retrieval. In addition, the PRNU-based calibration of CCD response non-uniformity, which is not addressed in Refs. [9,10,11], further improves the reliability of spatial temperature mapping across the image plane [12]. Experimental validation presented in later sections demonstrates that these design advantages translate into a reconstruction accuracy within 3.3% relative error compared to conventional spectral inversion, with a temporal resolution of 1 ms and a spatial mapping uncertainty (PRNU) below 2.2%.

## 2. Method

### 2.1. Spectral Thermometry and Temperature Element Distribution Probability Theory

Deflagration fireballs generate extremely high temperatures and exhibit complex spectral characteristics during explosions. Precise spectral detection of these phenomena is crucial, as it enables the inversion of temperature distributions and thermal radiation properties from the fireball’s emission spectra, providing essential data for assessing explosion damage effects [13].

Spectral detection of deflagration fireballs is grounded in Planck’s blackbody radiation law, which describes the energy distribution of an ideal blackbody at different temperatures and serves as the theoretical foundation for temperature inversion [14]. By measuring the radiation intensity of a fireball within specific wavelength ranges, spectral unmixing techniques can decompose the measured spectrum into multiple temperature components and calculate their proportional contributions within the detection area [15]. This approach not only captures temperature information but also facilitates analysis of radiative properties and energy distribution [16]. According to Planck’s law [17],

(1) $$B(\lambda ,T)=\frac{2hc^{2}}{\lambda^{5}}\left(\frac{1}{e^{hc/\lambda k_{B}B^{T}}-1}\right)$$

where B(λ,T) is the spectral radiant exitance ($W\cdot m^{-3}$), h is the Planck constant ($6.626\times 10^{-34}\;J\cdot s$), c is the speed of light ($3\times 10^{8}\;m/s$) λ is the wavelength (m), $k_{B}$ is the Boltzmann constant ($1.381\times 10^{-23}\;J/K$), and T is the absolute temperature (K).

### 2.2. Nonlinear Least Squares Fitting for Temperature Element Distribution

The combined effects of conduction, convection, and radiation tend to homogenize the temperature distribution in high-temperature objects [18]. Thus, the temperature distribution can be assumed to vary continuously and be represented as a discrete sequence of temperature elements within the field of view [19] For example, after determining the maximum and minimum temperatures of the deflagration fireball using the Wien transform, temperature elements are constructed at 50 K intervals across the identified temperature range [20]. Each temperature element corresponds to an elemental radiation spectrum derived from the blackbody radiation law [21]. The primary objective of the fitting process is to iteratively adjust the weight coefficients of these temperature elements until the synthesized spectrum optimally matches the measured spectrum [22].

### 2.3. Radiation Spectral Theoretical Model Based on Temperature Elements

According to Planck’s law, the radiance of a deflagration field can be expressed as follows:

(2) $$L(\lambda ,T)=\varepsilon (\lambda )\frac{c_{1}}{\pi \lambda^{5}}\frac{1}{e^{\frac{c_{2}}{\lambda T}}-1}$$

where $\varepsilon \left(\lambda \right)$ is the emissivity, λ is the wavelength, and T is the temperature. The first radiation constant is defined as $c_{1}=2\pi hc^{2}=\frac{\left(3.7415\pm 0.0003\right)\times 10^{-16}\;w}{m^{2}}$, and the second radiation constant is $c_{2}=\frac{hc}{k}=\frac{\left(1.43879\pm 0.00019\right){\times 10}^{-2}\;m}{K}$.

In practical deflagration fireballs, the radiation source deviates from an ideal blackbody. Although the emissivity term ε(λ) is included in Equation (2), the emissivity of soot particles, combustion products, and condensed-phase fragments depends on wavelength, temperature, and spatial position. Moreover, the radiation emitted from the fireball may be absorbed or scattered during propagation through incandescent gas, smoke, and combustion products. Accordingly, the measured spectral signal is more generally expressed as follows [23]:

(3) $$S_{\mathrm{meas}}(\lambda )=R(\lambda )\left[\tau_{g}(\lambda )\sum_{i=1}^{N}\alpha_{i}\varepsilon_{i}(\lambda ,T_{i})B(\lambda ,T_{i})+L_{g}(\lambda )\right]+n(\lambda )$$

where $S_{\mathrm{meas}}(\lambda )$ is the measured spectral signal, R(λ) is the instrument response function, $\tau_{g}(\lambda )$ is the effective spectral transmittance of incandescent gas and smoke along the optical path, $\alpha_{i}$ is the weight coefficient of the *i*-th temperature element, $\varepsilon_{i}(\lambda ,T_{i})$ is the effective emissivity of the corresponding temperature element, $B(\lambda ,T_{i})$ is the blackbody radiation intensity calculated from Planck’s law, $L_{g}(\lambda )$ represents the self-emission contribution of hot gas along the optical path, and n(λ) denotes measurement noise.

For transient deflagration fireballs, a spectral pyrometry system was adopted to reconstruct the average emissivity and average temperature simultaneously. In this study, the wavelength-dependent emissivity of combustion products and the gas transmittance were not independently measured. Therefore, temperature reconstruction was performed under an effective graybody approximation after instrument response correction and spectral normalization. Under this approximation, the effects of emissivity and gas transmission are partially subsumed into the fitted temperature-element weights and the normalized spectral shape. However, if the emissivity or gas transmittance varies significantly across the measured spectral range, the fitted spectrum may deviate from the actual radiation spectrum, introducing additional uncertainty into the temperature reconstruction. The quantitative bounds of this deviation are discussed in the error analysis section.

The radiant flux $\phi \left(\lambda ,T\right)$ received at the fiber end face of the spectrometer can be expressed as follows:

(4) ϕ(λ,T)=L(λ,T)·ΔS·Sr

ΔS denotes a surface element on the radiation surface of the deflagration field. Its size is determined by the object–image relationship and the fiber end face dimensions. Sr represents the solid angle subtended by the surface element ΔS to the lens.

**Figure 1:**
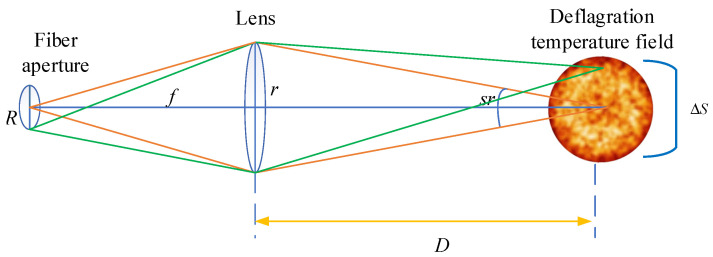
Schematic diagram of the optical imaging system for capturing light from an explosion fireball.

As shown in Figure 1, the area of the surface element ΔS is given by the following:

(5) $$\Delta S={\pi \left(\frac{DR}{f}\right)}^{2}$$

This system comprises an explosion fireball (as the light source), a lens, and an optical fiber with a core radius of R. Light emitted by the explosion fireball is imaged onto the fiber end face via the lens. Specifically, the light radiated from a single point in the fireball within a solid angle of Sr is captured by the lens and subsequently converged onto the fiber end face. Herein, D denotes the distance between the explosion fireball and the lens, f represents the focal length of the lens, and ΔS refers to the luminous area of the fireball captured by the fiber core.

In the formula, f is the focal length of the lens, and R is the radius of the optical fiber. When the lens radius r is much smaller than the object distance (r≪D), the radiative solid angle Sr of the surface element is given by the following:

(6) $$Sr=\frac{\pi r^{2}}{D^{2}+r^{2}}$$

where D is the distance from the temperature field to the lens, and r is the radius of the lens. Finally, the radiant flux function $\phi \left(\lambda ,T\right)$ is derived as follows:

(7) $$\phi \left(\lambda ,T\right)=L\left(\lambda ,T\right)\cdot \pi {\left(\frac{DR}{f}\right)}^{2}\frac{\pi r^{2}}{D^{2}+r^{2}}=L\left(\lambda ,T\right)\cdot {\left(\frac{\pi rR}{f}\right)}^{2}\cdot \frac{D^{2}}{D^{2}+r^{2}}$$

Assuming the deflagration field is composed of multiple temperature elements $T_{1},T_{2}{\ldots T}_{m}$, each corresponding to areas ${S_{1},S_{2}\ldots S}_{m}$, the radiant flux associated with each temperature element is expressed as follows:

(8) $$\phi \left(\lambda_{n},T_{m}\right)=L\left(\lambda_{n},T_{m}\right)\cdot \frac{D^{2}}{D^{2}+r^{2}}\cdot S_{m}$$

The contribution of each temperature element $T_{m}$ to the total radiant flux depends not only on its radiation intensity but also on its corresponding area $S_{m}$. Therefore, the weight coefficient $\omega_{T_{m}}$ of the temperature element $T_{m}$ can be expressed as the ratio of its area $S_{m}$ to the total area $S_{total}$:

(9) $$\omega_{T_{m}}=\frac{S_{m}}{S_{\mathrm{total}}}=\frac{S_{m}}{\pi {\left(\frac{D\cdot R}{f}\right)}^{2}}$$

In summary, the total radiant flux $\phi \left(\lambda_{n}\right)$ can be expressed as follows:

(10) $$\begin{array}{cc} \phi \left(\lambda_{n}\right) & =\sum_{m}\left(L\left(\lambda_{n},T_{m}\right)\cdot {\left(\frac{\pi \cdot r\cdot R}{f}\right)}^{2}\cdot \frac{D^{2}}{D^{2}+r^{2}}\cdot \omega_{T_{m}}\right) \end{array}$$

where $\omega_{T_{m}}$ represents the weight coefficient of each temperature element, and the sum of all weights satisfies the normalization condition: $\sum_{m}\omega_{T_{m}}=1$.

## 3. Objective Function

The fitting process is optimized by minimizing an objective function that quantifies the discrepancy between the measured spectrum and the fitted spectrum through their spectral slopes [24]. Specifically, the wavelength differentiation method is used to calculate the spectral slope, and the mean squared error (MSE) is employed to measure the difference between the slopes of the two spectra [25]. The MSE formula is defined as follows:

(11) $$\mathrm{MSE}=\frac{1}{n-1}\sum_{i=1}^{n-1}{\left(\frac{dI_{\mathrm{measured}}}{d\lambda_{i}}-\frac{dI_{\mathrm{fitted}}}{d\lambda_{i}}\right)}^{2}$$

where $I_{\mathrm{measured}}$ is the measured spectral intensity, $I_{\mathrm{fitted}}$ is the fitted spectral intensity, Δλ is the wavelength increment, and n is the total number of spectral data points. Slope optimization captures the global variation trend of the spectrum, avoiding overemphasis on local intensity differences. This approach effectively reduces the impact of noise, enhances fitting stability, and accurately reflects the contributions of different temperature elements [26].

The fitting model is based on Planck’s blackbody radiation law, where the measured spectrum is simulated as a linear combination of elemental radiation spectra corresponding to distinct temperature elements [27]. The expression for the fitted spectrum is as follows:

(12) $$I_{\mathrm{fitted}}(\lambda )=\sum_{i=1}^{m}w_{i}\cdot I_{\mathrm{Planck}}\left(\lambda ,T_{i}\right)$$

where $I_{\mathrm{fitted}}$ is the fitted spectrum, $w_{i}$ is the weight coefficient of the *i*-th temperature $T_{i}$, and $I_{\mathrm{Planck}}\left(\lambda ,T_{i}\right)$ is the Planck-derived spectral intensity at temperature $T_{i}$. By adjusting the weights $w_{i}$, a composite spectrum matching the measured data can be generated.

### 3.1. Initial Setup of Temperature Elements and Weight Coefficients

Prior to least squares optimization, establishing reasonable initial temperature elements and initial weight coefficients is critical to ensure a smooth optimization process and enhance fitting accuracy. Proper initialization not only provides a robust starting point for optimization but also helps avoid convergence to local optima.

1. Determination of Initial Temperature Elements

Initial temperature elements are determined through Wien transform of the spectral curve. Based on Planck’s law, the Wien transform analyzes experimentally measured spectral data to infer corresponding temperature elements.

2. Initialization of Weight Coefficients

Initial weight coefficients are derived from the mapping relationship between the grayscale value distribution of the image and temperature. The grayscale values in the image reflect the radiative intensity of individual pixels, and different intensity levels correspond to distinct temperatures. The initialization process involves the following steps:

- Grayscale value distribution analysis.

Statistical analysis of grayscale value distributions identifies the nonlinear relationship between grayscale values and temperature.

- Mapping function construction.

A grayscale-to-temperature mapping function is formulated based on the grayscale distribution characteristics.

- Initial weight assignment.

Using the mapping function, grayscale values in specific image regions are correlated with corresponding temperature elements. Initial weights are assigned to each temperature element, reflecting their relative contributions across the image.

3. Significance and Advantages of Initialization

The above initialization strategy provides a physically grounded and data-driven starting point for least squares optimization. Key advantages include the following:

- Mitigation of local optima.

Physically meaningful initial values enhance the global convergence of the optimization process.

- Improved fitting accuracy.

Initialization based on Wien transform and grayscale mapping ensures alignment with actual spectral characteristics, boosting result reliability.

- Reduced computational costs.

Appropriate initialization minimizes the number of iteration steps, significantly enhancing computational efficiency. By integrating spectral analysis with image data, this initialization framework ensures both efficiency and accuracy in reconstructing temperature fields.

### 3.2. Temperature Weight Optimization Method: Objective Function Minimization

To optimize the temperature weight coefficients, the limited-memory Broyden–Fletcher–Goldfarb–Shanno algorithm with bound constraints (L-BFGS-B) is employed [28]. This algorithm utilizes gradient information of the objective function to optimize parameters while enforcing boundary constraints, restricting the weights to the range [0, 1]. The optimization process iteratively adjusts the temperature weights to minimize the objective function, specifically the slope error [29]. Based on nonlinear least squares methodology, this approach combines slope-difference error minimization with the L-BFGS-B algorithm to refine temperature weights and achieve precise fitting of the measured spectrum. By integrating Planck’s law with statistical optimization techniques, it effectively decomposes composite spectra contributed by multiple temperature components, ultimately yielding optimal temperature weights and highly accurate fitting results.

### 3.3. Theory of Spectral Thermometry and Image Fusion

During temperature field reconstruction and spectral data processing, temperature values are assigned to grayscale levels through weighted averaging based on temperature elements and their distribution weights [30]. This mapping effectively integrates temperature information into grayscale images, producing pseudo-color temperature distributions.

1. Relationship Between Temperature Elements and Grayscale Images

The input grayscale image contains pixel grayscale values $G_{\left(x,y\right)}$, which represent the local radiation intensity. By correlating the weight coefficients of temperature elements with their temperature distributions, each pixel’s grayscale value is mapped to a corresponding temperature.

2. Grayscale Classification and Temperature Assignment

First, grayscale values in the image are classified. Low-grayscale regions (e.g., background areas) typically correspond to ambient temperatures or non-combustible zones. These regions exhibit low radiation intensity and do not reflect high-temperature components of the fireball. 

Therefore, low-grayscale regions are identified via a threshold $G_{low}$, and their temperatures are set to the minimum or ambient temperature. High-grayscale regions are mapped to temperature elements derived from spectral analysis.

(13) $$G_{\left(x,y\right)}<G_{low}\to T_{\left(x,y\right)}=T_{env}$$

where $T_{env}$ is the ambient temperature. Regions where $G_{\left(x,y\right)}<G_{low}$ are excluded from high-temperature assignments, and their temperature is directly set to $T_{env}$. High-grayscale regions correspond to high-temperature zones. The temperature assignment for grayscale values $G_{\left(x,y\right)}$ follows these steps: Divide the grayscale values into multiple intervals based on the weight ranges of the temperature elements, with each interval corresponding to a specific temperature element. Let $G_{\min}$ be the minimum grayscale value and $G_{\max}$ the maximum grayscale value. The grayscale values in the image are segmented according to the weight $\omega_{i}$ of the temperature elements, ensuring the proportion of grayscale values corresponds to $\omega_{i}$.

For the i-th temperature element $T_{i}$, the corresponding grayscale range is $\left[G_{\min_{i}},G_{\max_{i}}\right]$, and the weight *α* can be expressed by Equation (13).

(14) $$w_{i}=\frac{{\mathrm{Area}}_{i}}{\mathrm{Total}\;\mathrm{Area}}=\frac{\int_{G_{\min_{i}}}^{G_{\max_{i}}}p(G)dG}{\int_{G_{\min}}^{G_{\max}}p(G)dG}$$

where p(G) is the probability density function (PDF) of the grayscale values in the image, representing the distribution of grayscale values and indicating the frequency of occurrence for each grayscale level. ${\mathrm{Area}}_{i}$ denotes the proportion of the grayscale interval corresponding to the temperature element, and Total Area represents the total proportion of grayscale distribution in the image, typically normalized to 1. $\int_{G_{\min_{i}}}^{G_{\max_{i}}}p(G)dG$ represents the cumulative probability within the grayscale interval $\left[G_{\min_{i}},G_{\max_{i}}\right]$, which is proportional to the area proportion $\omega_{i}$ associated with the temperature element $T_{i}$.

Through this approach, the weight coefficient $\omega_{i}$ of the temperature element is aligned with the distribution of grayscale ranges in the image, ensuring consistent temperature assignment relative to the grayscale distribution.

## 4. Spectral-Image Fusion Rapid Detection Device

### Device Development

The detection system consists of three core components, which are laid out as the experimental setup shows: a CCD sensor, a fiber collimator and a fiber-optic spectrometer. The fiber collimator is integrated with the CCD sensor on a shared support, and their optical axes have been carefully adjusted to be parallel. The detection system consists of a CCD camera, an imaging lens, a fiber collimator, a visible-near-infrared quartz fiber, a fiber-optic spectrometer (Model USB2000+, Ocean Optics/Ocean Insight, Dunedin, FL, USA), and a computer for data acquisition and processing. Table 1 summarizes the models and key parameters of the main hardware components. The CCD camera (Model MV-CA003-21UM, Hikvision, Hangzhou, China) is an industrial area-scan camera equipped with an OnSemi PYTHON300 CCD sensor (onsemi, Scottsdale, AZ, USA), offering a resolution of 640 × 480 pixels, a pixel size of 4.8 μm × 4.8 μm, a spectral response range of 300–1100 nm, and a maximum frame rate of 814.5 fps at full resolution. In the experiment, both the CCD camera and the fiber-optic spectrometer were operated at 340 fps with an integration time of 1 ms, achieving millisecond-resolution synchronous acquisition of image and spectral data.

As listed in Table 1, both the fiber-optic spectrometer and the CCD sensor were operated with an integration time of 1 ms and a frame rate of 340 fps, yielding a theoretical acquisition interval of 1/340 ≈ 2.94 ms. Four consecutive frames at 189, 192, 195, and 198 ms after ignition were selected for temperature reconstruction, corresponding to a sampling interval of approximately 3 ms. These results demonstrate that the proposed spectral-image fusion system can resolve the transient temperature evolution of deflagration fireballs with a temporal resolution of approximately 2.94 ms.

**Figure 2:**
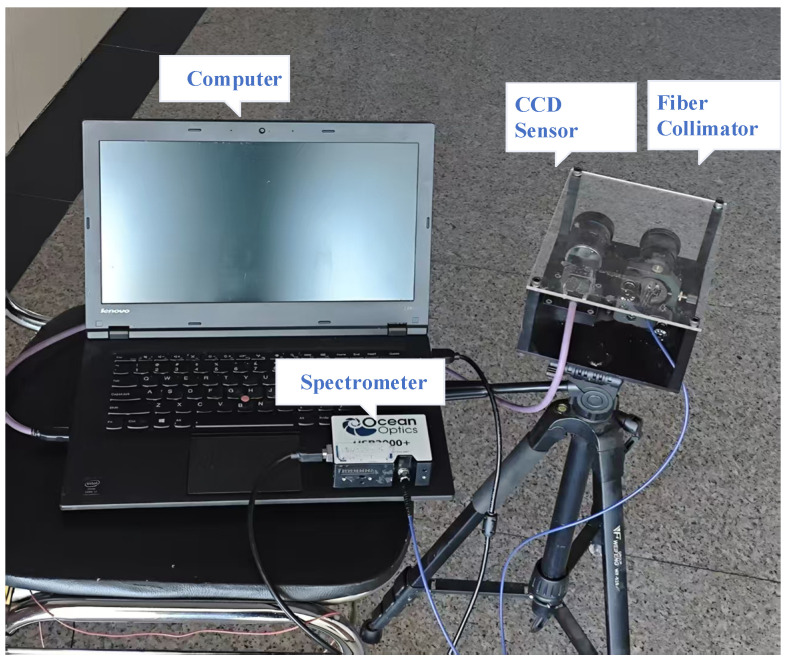
Experimental setup of the detection system, which includes a computer used for data transmission, a CCD sensor, a fiber collimator, and a fiber-optic spectrometer. The fiber collimator is integrated with the CCD sensor on a shared support fixed to a tripod, and all components are connected via cables to enable data interaction.

The physical structure and component layout of the detection system are illustrated in Figure 2. This system is mainly composed of a data transmission computer, a CCD sensor, a fiber collimator and a fiber-optic spectrometer. The fiber collimator and CCD sensor are mounted on a unified bracket fixed to a tripod, and all devices are connected by cables for real-time data transmission and in-teraction.

## 5. Spectral Calibration

The fiber-optic spectrometer was radiometrically calibrated before the deflagration experiment using a standard blackbody radiation source (DY-HT3, temperature range of 300–1200 °C, emissivity ≥ 0.995, and resolution of 0.1 °C, Detu Precision Instruments, Changzhou, China). The calibration system comprised the blackbody furnace, optical fiber, spectrometer, and data acquisition computer. The blackbody was heated stepwise from 800 K to 1200 K at intervals of 50 K, and each temperature point was maintained for 30 min to ensure thermal equilibrium and minimize the influence of transient temperature drift on the detector response. 

At each temperature point, the radiation spectrum emitted by the blackbody was recorded by the spectrometer. The corresponding theoretical blackbody spectrum was calculated using Planck’s law. By comparing the measured spectra with the theoretical spectra, the wavelength-dependent instrument response function was obtained. This response function characterizes the combined photoelectric conversion efficiency and spectral sensitivity of the optical fiber, spectrometer, and detector, and was subsequently used to correct the raw spectra acquired from the deflagration fireball.

It should be noted that a prominent peak appears in the short-wavelength region of the calibration curve. This peak does not correspond to a physical radiation peak of the blackbody source or the deflagration fireball. In the short-wavelength region, the blackbody radiation intensity is relatively low and the combined sensitivity of the optical fiber, spectrometer, and detector is also low. Consequently, the measured signal has a low signal-to-noise ratio, and the calculated correction coefficient may be amplified by dark noise, stray light, and detector response uncertainty. Therefore, the short-wavelength portion of the calibration curve is less reliable for quantitative temperature fitting.

To mitigate this issue, temperature inversion and spectral fitting were performed in the spectral region with more stable signal quality. In this study, the fitting was primarily conducted over the 500–700 nm range. The short-wavelength features in the calibration curve were used only to characterize the instrument response and were not treated as temperature-related spectral information.

**Figure 3:**
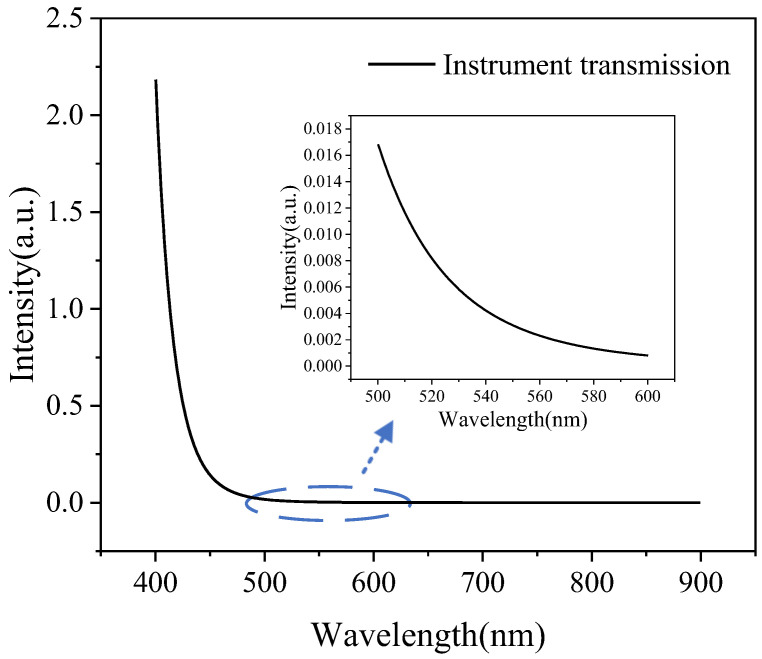
Instrument response function of the spectrometer.

Calibration Procedure: Blackbody Heating: The blackbody is heated to a known temperature, emitting a spectrum that conforms to Planck’s radiation law. Spectral Recording: The spectrometer measures the radiation intensity across wavelengths under controlled conditions. Response Function Calculation: Using the blackbody’s known radiation properties, the instrument response function—which represents the spectrometer’s wavelength-dependent efficiency—is derived. The spectrometer’s instrument response function is shown in Figure 3, highlighting its response characteristics across wavelengths. Post-calibration, the instrument response function is applied to correct raw spectral data by eliminating systematic instrument errors, resulting in reliable spectral curves that accurately represent the radiation properties of the deflagration fireball. These calibrated data serve as the foundation for subsequent temperature inversion analysis.

### Numerical Validation Using Simulated Standard Blackbody Spectra

To evaluate the reliability of the spectral inversion procedure under controlled conditions, a simulated validation was performed using standard blackbody radiation spectra. Blackbody spectra at 800, 900, 1000, 1100, and 1200 K were used to construct the instrument-response correction model, while the intermediate temperatures of 850, 950, 1050, and 1150 K were reserved as validation points and excluded from the response-function fitting. The simulated spectra were processed using the same calibration, smoothing, normalization, and nonlinear least-squares fitting procedure applied to the deflagration fireball spectra. The relative error of the blackbody validation is defined as follows:

(15) $$\delta_{BB}=\frac{\left|T_{\mathrm{set}}-T_{\mathrm{retrieved}}\right|}{T_{\mathrm{set}}}\times 100\%$$

where $T_{\mathrm{set}}$ is the set temperature of the standard blackbody source, and $T_{\mathrm{retrieved}}$ is the temperature retrieved by the proposed spectral reconstruction method.

The validation results are listed in Table 2. The retrieved temperatures at 850, 950, 1050, and 1150 K were 858, 941, 1061, and 1138 K, corresponding to relative errors of 0.9%, 0.8%, 1.0%, and 1.0% (average 0.9%). These results confirm that the proposed spectral inversion procedure recovers blackbody radiation temperatures with an average error below 1% under idealized conditions. However, this simulated validation does not fully capture the uncertainties in actual deflagration measurements, where emissivity variation, gas absorption, smoke scattering, and other field-specific factors may introduce additional errors.

## 6. CCD Response Characteristics and PRNU Calculation

To ensure accurate characterization of the CCD’s response across varying distances, we employ the PRNU calculation method. PRNU quantifies the response variation among pixels under uniform illumination conditions, reflecting the sensor’s inherent spatial non-uniformity.

(16) $$\mathrm{PRNU}=\frac{1}{N}\sum_{i=1}^{N}{\left(\frac{I_{i}-\mu }{\mu }\right)}^{2}$$

where $I_{i}$ is the response value of the i-th pixel, μ is the mean value of the responses, and N is the number of measured pixels.

In the experiment, the MV-CA003-21UM CCD camera (OnSemi PYTHON300, 640 × 480, 4.8 μm pixel pitch, 300–1100 nm spectral range) was used to image a medium-temperature blackbody furnace at a distance of 9 m. The camera was operated at 340 fps with an integration time of 1 ms, consistent with the deflagration measurement settings. The calculated PRNU value was below 2.2%, confirming that the grayscale distribution across the sensor provides sufficiently uniform spatial information for subsequent temperature-field reconstruction.

**Figure 4:**
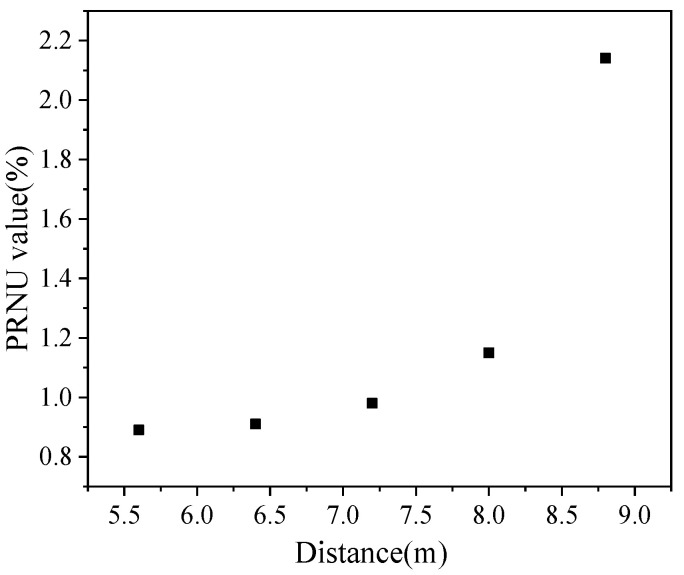
PRNU values of the CCD sensor.

Leveraging the quantitative data presented in Figure 4, the distance-dependent variation characteristics of the CCD’s PRNU are analyzed as follows: within the range of 5.5 m to 8.0 m, PRNU values stabilize between 0.8% and 1.2% with negligible fluctuations, and from the physical perspective of PRNU, this behavior implies that the CCD exhibits only minor inter-pixel response discrepancies under uniform illumination within this distance interval—reflecting exceptional photo-response uniformity that guarantees robust inter-pixel consistency for light intensity measurements. In contrast, PRNU values surge sharply to approximately 2.2% at 8.75 m and beyond, signifying a substantial deterioration in photo-response uniformity attributed to the enhanced inter-pixel response variations.

### Influence of Hardware Replacement on Measurement Error

The measurement error of the proposed spectral-image fusion method is hardware-dependent. In this study, hardware replacement experiments were not conducted; therefore, the exact error bounds for alternative hardware configurations cannot be directly provided. However, the following analysis outlines the factors that would affect the error and the procedure for re-evaluation after any component change.

Replacement of any optical component—CCD sensor, spectrometer, lens, fiber collimator, or acquisition system—requires full recalibration, because the spectral response, photo-response non-uniformity, dynamic range, noise level, field of view, optical alignment, and synchronization accuracy may differ from the present configuration. A CCD with a higher PRNU or lower dynamic range increases the uncertainty in grayscale-to-temperature mapping; a spectrometer with a different wavelength response or lower signal-to-noise ratio affects the accuracy of temperature-element fitting. Changes in focal length, aperture, fiber core diameter, or optical-axis alignment can alter the spatial correspondence between the spectrometer detection area and the CCD image, thereby shifting the reconstructed temperature distribution.

Therefore, the relative errors reported in this paper are valid only for the present hardware under the current calibration conditions. After any hardware change, the spectrometer response function, CCD PRNU, geometric alignment, exposure parameters, and time synchronization must be recalibrated. The error of the new configuration can be evaluated by comparing the reconstructed temperature against a reference (a calibrated source or conventional spectral inversion) at multiple temperature points:

(17) $$\delta_{\mathrm{new}}=\frac{\left|T_{\mathrm{ref}}-T_{\mathrm{new}}\right|}{T_{\mathrm{ref}}}\times 100\%$$

where $T_{\mathrm{ref}}$ is the reference temperature and $T_{\mathrm{new}}$ is the temperature obtained with the new hardware.

## 7. Acquisition and Processing of Deflagration Fireball Spectral Data

### 7.1. Spectral Data Detection of Deflagration Fireball

**Figure 5:**
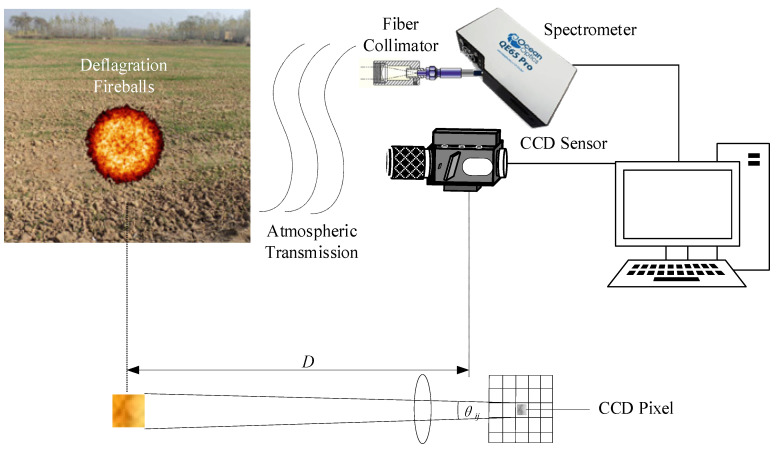
Schematic diagram of the experimental setup for temperature measurement of a deflagration fireball. This system utilizes a CCD sensor and a fiber-optic spectrometer to capture the image and emission spectrum, respectively, of a deflagration fireball positioned at a distance D from the detection assembly.

As shown in Figure 5, this system utilizes a CCD sensor and a fiber-optic spectrometer to capture the image and emission spectrum, respectively, of a deflagration fireball. Light emitted by a small luminous surface element within the deflagration fireball is focused onto a single pixel of the CCD through the imaging system. The output intensity of this CCD pixel is proportional to the integral of photon energy contributions from the small surface element, integrated over the full wavelength range. Correspondingly, the spectral intensity output by the fiber-optic spectrometer at each individual wavelength scales proportionally to the radiant intensity of the small surface element at that respective wavelength.

**Figure 6:**
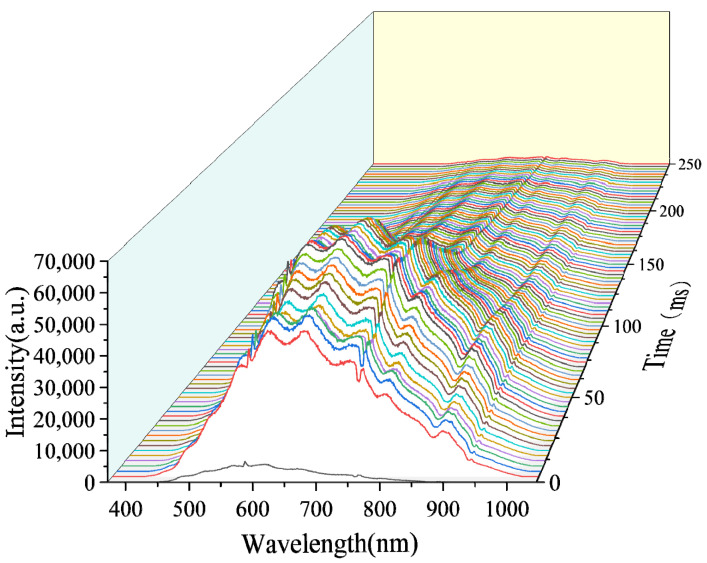
Raw spectra of deflagration fireball.

The experiment was conducted in December 2023, under local ambient conditions of 1 °C and 70% humidity. A spectrometer and CCD sensor were positioned 200 m from the detonation point to synchronously monitor the deflagration fireball. The raw spectral data of the fireball are shown in Figure 6.

Figure 6 presents a waterfall plot of the raw spectra of the deflagration fireball over the time interval of 0 to 250 ms. The horizontal axis represents the wavelength detected by the spectrometer from 372.05 nm to 1047.04 nm, the vertical axis indicates the raw spectral intensity of the deflagration fireball, and the *Z*-axis depicts the temporal evolution of the fireball spectrum. Data indicate that the spectral intensity rises sharply within 0–30 ms, drops sharply between 30 and 50 ms, and decreases slowly from 50 to 250 ms.

### 7.2. Processing of Deflagration Fireball Spectral Data

First, the raw measured spectral data are calibrated for responsivity based on the intensity response characteristics of the instrument. Subsequently, the calibrated spectral data undergo smoothing filtering to mitigate noise, followed by normalization to standardize the data amplitude. Next, the temperature distribution range of the target is derived via Wien’s transformation, and a set of temperature primitives are constructed accordingly. Taking the theoretical blackbody radiation data of each temperature primitive and their corresponding distribution weights as input parameters, nonlinear least-squares fitting is performed by fusing the fiber-optic spectrometer data and CCD sensor data. In this fitting process, the weight of each temperature primitive corresponding to the optimal solution correlates with the distribution weight of the CCD sensor—specifically, the grayscale histogram of the CCD image—thereby establishing a quantitative mapping between the CCD grayscale values and the pre-constructed temperature primitives. Ultimately, the integrated processes of spectral temperature retrieval and image fusion are accomplished through this systematic workflow.

**Figure 7:**
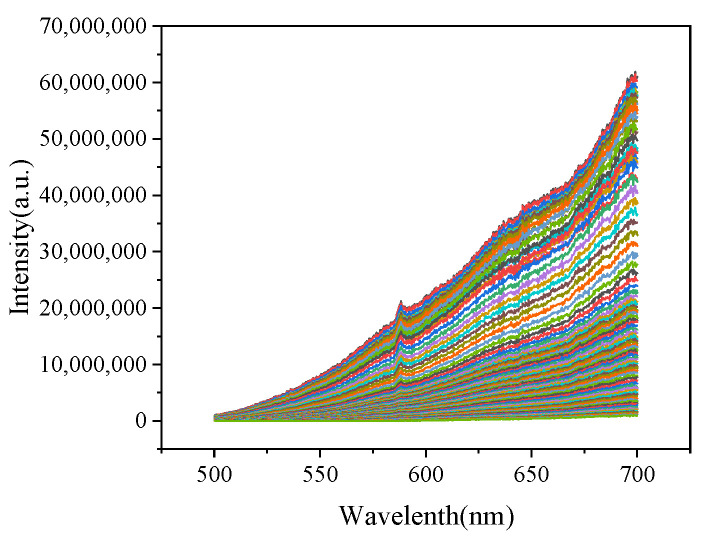
Instrument response function corrected spectral curve of the deflagration fireball.

The true corrected spectral curve of the deflagration fireball, obtained after instrument response function calibration of the original spectral data, is shown in Figure 7.

Figure 7 presents the true spectral curves of the deflagration fireball after correction for the instrumental transfer function. The horizontal axis represents the visible wavelength range from 500 to 700 nm, while the vertical axis indicates the radiant intensity. The curves exhibit a significant increase with wavelength, particularly pronounced in the long-wavelength visible region. Multiple curves correspond to different deflagration conditions. These corrected data eliminate instrumental deviations and provide a reliable foundation for subsequent smoothing, normalization, and fitting of the theoretical model using the nonlinear least squares method to retrieve the deflagration fireball temperature. They clearly reflect the true visible light radiation characteristics of the deflagration fireball and constitute a crucial step in the spectral temperature retrieval process.

### 7.3. Temperature Inversion of Deflagration Fireball Spectrum

**Figure 8:**
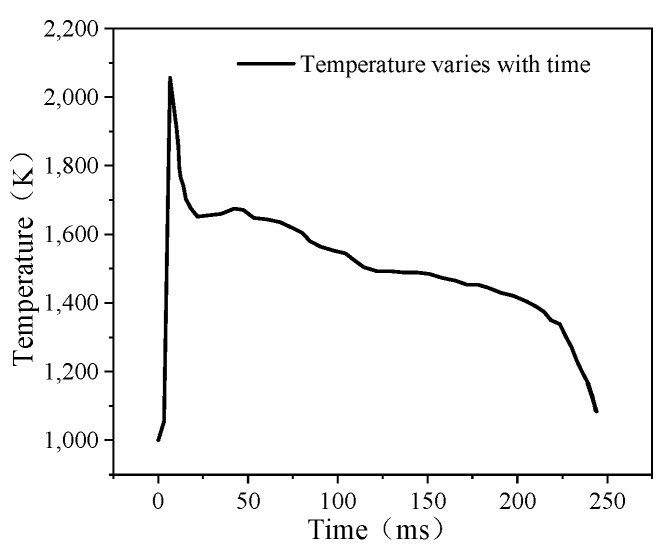
Temperature–time profile of the deflagration fireball.

As shown in Figure 8, the temperature–time variation curve was obtained by inverting the data from Figure 7 via spectral temperature inversion technology.

Figure 8 exhibits the temporal evolution characteristics of the deflagration fireball temperature: At 6 ms, the temperature rises rapidly to 2050 K, followed by a sharp decrease to 1650 K. It then increases slightly to 1670 K at 39 ms before undergoing a continuous cooling process, with the temperature dropping below 1050 K by 240 ms. As shown in the temperature–time curve, the temperatures at 189 ms, 192 ms, 195 ms, and 198 ms are approximately 1430 K, 1422 K, 1414 K, and 1406 K, respectively.

The maximum temperature captured by spectral inversion in the present experiment was approximately 2050 K at 6 ms (Figure 8). This value represents the maximum observed under the current experimental conditions rather than the absolute upper measurement limit of the system. The measurable range is determined by the calibrated spectral bandwidth, detector dynamic range, integration time, signal-to-noise ratio, and the onset of spectral or image saturation. To measure higher-temperature fireballs, the integration time, neutral-density attenuation, and calibration range must be adjusted accordingly to avoid detector saturation and maintain reliable temperature inversion.

**Figure 9:**
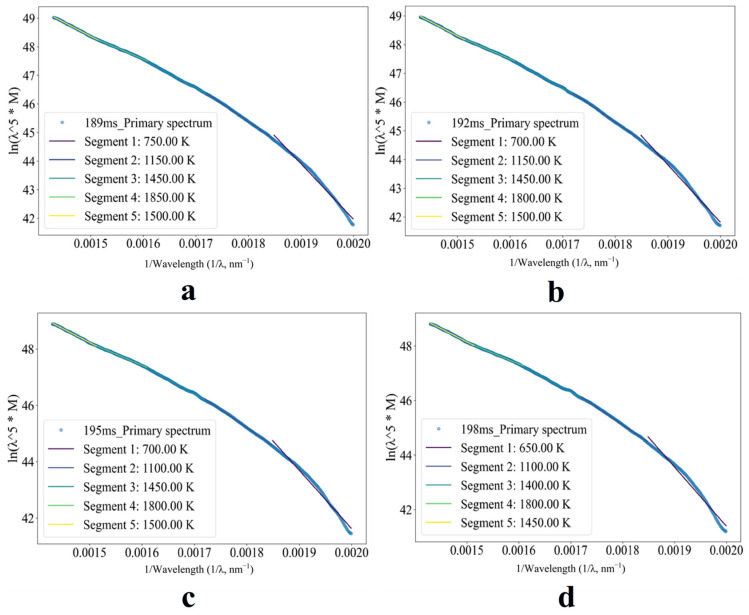
Temperature ranges derived from Wien transform: (**a**) 189 ms temperature range; (**b**) 192 ms temperature range; (**c**) 195 ms temperature range; and (**d**) 198 ms temperature range. (The Primary spectrum in the figure has a high data density with points connected into lines).

The temperature evolution curve, derived from Wien’s transform calculations at four selected time points—189 ms, 192 ms, 195 ms, and 198 ms—is depicted in Figure 9. The results indicate a temperature range of 650 K to 1850 K during this period.

**Figure 10:**
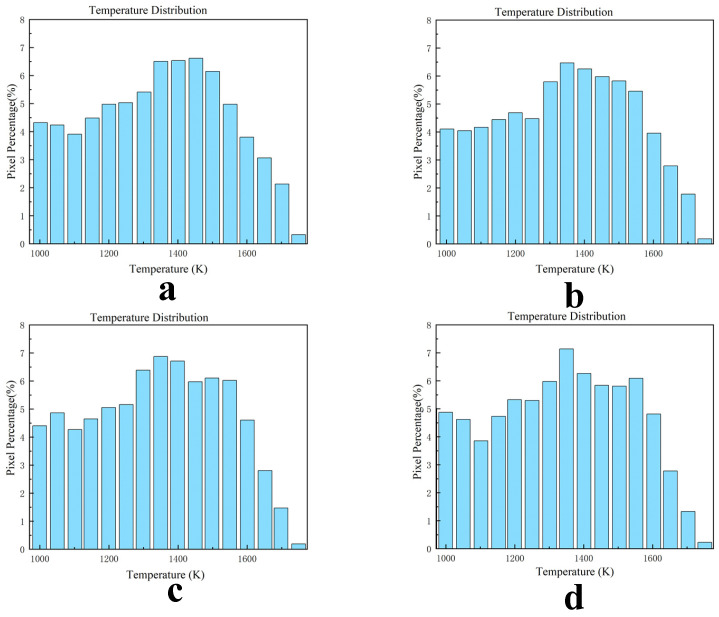
Initial temperature weight distributions derived from grayscale values: (**a**) 189 ms initial weights; (**b**) 192 ms initial weights; (**c**) 195 ms initial weights; and (**d**) 198 ms initial weights.

The Stefan–Boltzmann law establishes the quantitative relationship between the total radiant exitance of an ideal blackbody across all wavelengths and its temperature, expressed as follows:

(18) $$M^{0}={\sigma \cdot T}^{4}$$

where $M^{0}$ is the total radiant exitance, and σ is the Stefan–Boltzmann constant. This law demonstrates that the radiant exitance of a blackbody is proportional to the fourth power of its absolute temperature. Based on this $T^{4}$ relationship between radiant exitance and temperature, the initial temperature weight distribution for the least squares calculation was determined. The initial temperature weight distributions derived from grayscale values at 189 ms, 192 ms, 195 ms, and 198 ms are shown in Figure 10.

**Figure 11:**
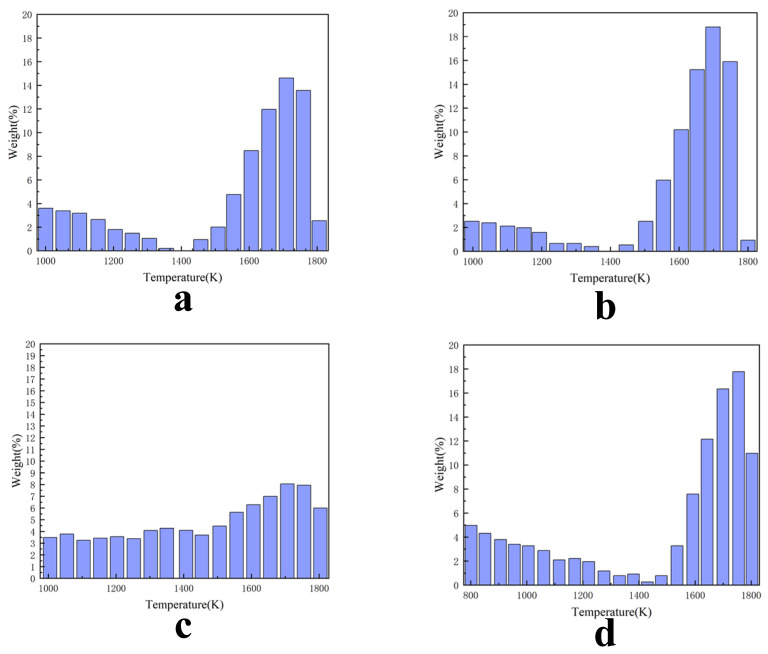
Temperature inversion results of the deflagration fireball: (**a**) 189 ms temperature inversion results; (**b**) 192 ms temperature inversion results; (**c**) 195 ms temperature inversion results; and (**d**) 198 ms temperature inversion results.

Based on the determined temperature ranges, fitting intervals with a temperature step of 50 K were established. Through inversion, the final derived temperatures and their proportional distributions are shown in Figure 11.

### 7.4. Comparison with Conventional Spectral Inversion and Alternative Methods

To evaluate the reliability of the proposed spectral-image fusion method, the reconstructed average temperatures were compared with those obtained by conventional spectral inversion at four consecutive time points. The relative error is defined as follows:

(19) $$\mathrm{Relative}\;\mathrm{Error}=\frac{\left|T_{spectral}-T_{fitted}\right|}{T_{spectral}}\times 100\%$$

where $T_{spectral}$ represents the temperature obtained by conventional spectral inversion, $T_{fitted}$ denotes the average temperature obtained by the proposed fitting method, and $T_{spectral}-T_{fitted}$ is the absolute temperature difference between the two methods.

The comparison results are listed in Table 3. At 189, 192, 195, and 198 ms, the average temperatures reconstructed by the proposed method were 1382, 1373, 1366, and 1357 K, respectively. The corresponding conventional spectral inversion temperatures were 1430, 1422, 1414, and 1406 K, yielding relative errors of 3.2%, 3.4%, 3.3%, and 3.4% (average 3.3%). These results confirm that the proposed method achieves good consistency with conventional spectral inversion while additionally providing two-dimensional spatial temperature distribution information.

As listed in Table 4, quantitative temperature results obtained by the conventional spectral inversion and the proposed method are compared at four typical moments in the fireball attenuation stage. The relative errors at all four time points are below 3.5%, confirming that the proposed method maintains stable temperature reconstruction performance across the tested temperature range. These errors represent the discrepancy between the proposed fusion method and conventional spectral inversion, rather than the absolute measurement uncertainty of the system. The absolute uncertainty may be influenced by spectral calibration accuracy, radiation model assumptions, emissivity variation, CCD response non-uniformity, image registration, and time synchronization.

The quantitative error analysis in this study was performed at four frames in the attenuation stage (1430–1406 K). Two-dimensional temperature reconstruction and error comparison at the peak-temperature moment (2050 K at 6 ms) were not conducted, because the selected frames were located in the decay phase of the fireball. Consequently, the error at the maximum temperature cannot be directly evaluated from the current data. Future work should reconstruct peak-temperature frames and compare them against reference spectral inversion or calibrated blackbody data to assess the method’s performance at higher temperatures.

A detailed comparison of technical characteristics, applicable conditions and limitations among existing fireball temperature measurement methods is provided in Table 5. The proposed method advances conventional spectral thermometry from average or line-of-sight temperature inversion to two-dimensional temperature field reconstruction by fusing synchronized spectral and imaging data. Compared with image-only methods, it introduces spectral constraints and does not rely solely on grayscale intensity for temperature inference. Compared with tomographic reconstruction approaches, it uses a simpler single-view experimental configuration, though its accuracy remains dependent on calibration quality, emissivity assumptions, gas transmission effects, and spectral-image registration.

### 7.5. Analysis of the Proposed Method

Although the relative errors between the fitted average temperatures and conventional spectral inversion are below 3.5% at all four time points, several potential error sources in the proposed spectral-image fusion method warrant consideration.

1. Spectral calibration and preprocessing

The instrument response function was obtained using a standard blackbody source, but wavelength drift, detector response non-uniformity, stray light, and residual calibration uncertainty may still affect the corrected spectral intensity. In addition, spectral smoothing suppresses random noise but may alter local spectral features, and normalization can weaken absolute intensity information linked to radiation energy.

2. Emissivity and radiative transfer

In the present fitting procedure, the radiation spectrum of each temperature element was approximated by Planck’s blackbody law, and the wavelength-dependent emissivity of combustion products was not independently measured. In actual deflagration fireballs, soot particles, gas species, and condensed-phase products exhibit wavelength-dependent emissivity, and the emitted radiation may be absorbed, scattered, or re-emitted by incandescent gas and smoke along the optical path. These effects distort the measured spectral shape and may introduce bias into the fitted temperature-element weights. Neglecting emissivity variation and gas transmission can lead to additional reconstruction error, particularly when strong spectral absorption or emission bands are present.

3. Temperature-element construction and spectral fitting

The temperature range is determined using Wien’s displacement law, and the temperature elements are discretized at a fixed interval. The selected range and step size therefore influence the final fitted weights. The nonlinear least-squares fitting may also be sensitive to the initial weight estimates and the objective function, especially when distinct temperature distributions produce similar spectral shapes.

4. Image-related errors

Although the PRNU method was used to characterize the imaging sensor response, residual pixel-response non-uniformity, exposure settings, saturation, lens vignetting, image noise, spatial registration mismatch between the imaging field and the spectrometer field of view, and temporal synchronization error between spectral and image acquisition can all affect the grayscale-to-temperature mapping.

**Figure 12:**
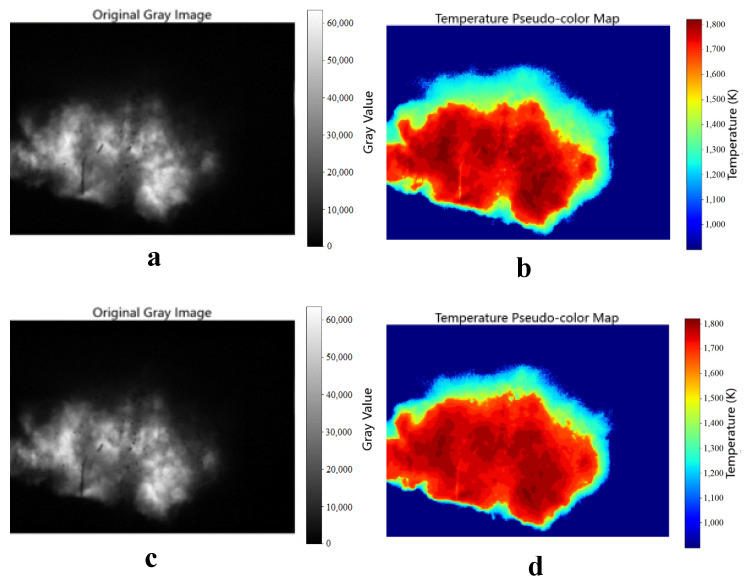

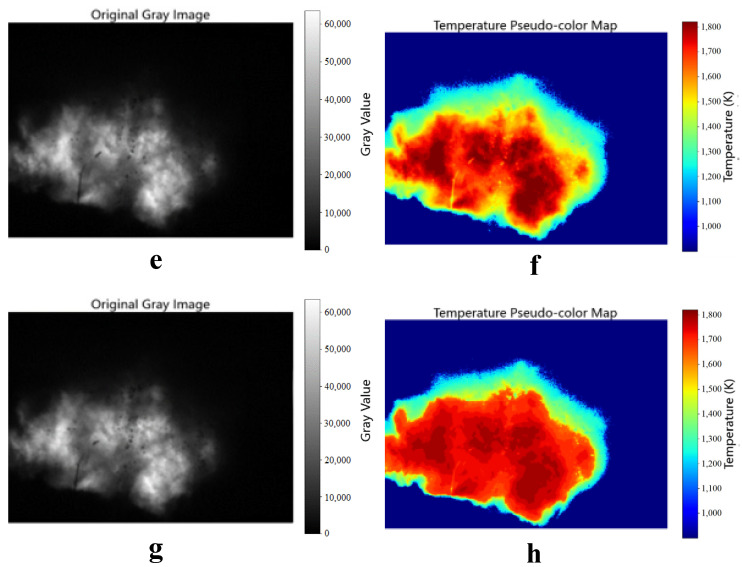
CCD Images and 2D temperature maps of deflagration fireball: (**a**,**b**) 189 ms deflagration fireball; (**c**,**d**) 192 ms deflagration fireball; (**e**,**f**) 195 ms deflagration fireball; and (**g**,**h**) 198 ms deflagration fireball.

As shown in Figure 12, the CCD images and corresponding two-dimensional temperature distribution of the deflagration fireball at four moments are presented. In this study, the effects of emissivity variation and gas transmission were treated as part of the effective spectral response and partially mitigated by instrument response correction and spectral normalization, but they were not explicitly decoupled. Consequently, the reported relative errors (Section 7.4) primarily reflect the consistency between the proposed method and conventional spectral inversion, rather than the absolute measurement uncertainty of the system. Future work will address this limitation by introducing emissivity correction, gas-transmission modeling, calibration under controlled flame conditions, and more accurate spectral-image registration.

### 7.6. Temperature Measurement Speed Evaluation

The measurement speed of the proposed system comprises two components: data acquisition speed and temperature reconstruction speed. The data acquisition speed is governed by the frame rates of the fiber-optic spectrometer and the CCD sensor. In the present configuration, both sensors were operated at 340 fps, yielding a theoretical acquisition interval of approximately 2.94 ms. This interval is consistent with the four consecutive frames selected at 189, 192, 195, and 198 ms, where the inter-frame spacing is approximately 3 ms. Thus, the system achieves millisecond temporal resolution for synchronized spectral-image acquisition.

The key parameters of the measurement speed for the proposed system are presented in Table 6. The temperature reconstruction process—including spectral calibration, smoothing, normalization, temperature-element construction, nonlinear least-squares fitting, and CCD grayscale mapping—was performed as post-processing of the acquired data in this study. Accordingly, the measurement speed reported here refers primarily to the temporal resolution of synchronized acquisition rather than real-time reconstruction. Future work will optimize the reconstruction algorithm and evaluate its computation time to enable real-time temperature field reconstruction.

### 7.7. Uncertainty Analysis of Temperature Measurement

A full uncertainty evaluation of the proposed spectral-image fusion method includes both spectrometer-related and imaging-camera-related contributions. The spectrometer governs the accuracy of spectral temperature inversion, while the imaging camera determines the spatial mapping of temperature elements onto the two-dimensional fireball image.

1. Spectrometer-related uncertainty

The main sources include blackbody calibration uncertainty $u_{\mathrm{cal}}$, wavelength calibration error $u_{\lambda }$, detector noise and dark current $u_{\mathrm{noise}}$, stray light and instrument response correction $u_{\mathrm{resp}}$, spectral smoothing and normalization $u_{\mathrm{pre}}$, emissivity approximation $u_{\varepsilon }$, gas-transmission effects $u_{\tau }$, and nonlinear fitting uncertainty $u_{\mathrm{fit}}$. These factors jointly affect the corrected spectral shape and the fitted temperature-element weights. The combined uncertainty of the spectrometer-based temperature inversion is expressed as follows:

(20) $$u_{\mathrm{spec}}=\sqrt{u_{\mathrm{cal}}^{2}+u_{\lambda }^{2}+u_{\mathrm{noise}}^{2}+u_{\mathrm{resp}}^{2}+u_{\mathrm{pre}}^{2}+u_{\varepsilon }^{2}+u_{\tau }^{2}+u_{\mathrm{fit}}^{2}}$$

In this study, $u_{\mathrm{spec}}$ = 2.1%, indicating that the spectral temperature inversion achieves high accuracy and provides a stable reference for the subsequent fusion algorithm.

2. Camera-related uncertainty

The main sources include photo-response non-uniformity $u_{\mathrm{PRNU}}$, image noise $u_{\mathrm{img}}$, exposure and gain settings $u_{\exp}$, saturation $u_{\mathrm{sat}}$, lens vignetting $u_{\mathrm{vig}}$, spatial registration error between the spectrometer field of view and the image field $u_{\mathrm{reg}}$, and temporal synchronization error $u_{\mathrm{sync}}$. The combined camera-related temperature-mapping uncertainty is as follows:

(21) $$u_{\mathrm{cam}}=\sqrt{u_{\mathrm{PRNU}}^{2}+u_{\mathrm{img}}^{2}+u_{\exp}^{2}+u_{\mathrm{sat}}^{2}+u_{\mathrm{vig}}^{2}+u_{\mathrm{reg}}^{2}+u_{\mathrm{sync}}}$$

The PRNU value of the imaging camera was experimentally evaluated and found to be less than 2.2% at a distance of 9 m, indicating acceptable photo-response uniformity under the present conditions. Considering all camera-related sources, the combined temperature-mapping uncertainty of the imaging camera was calculated to be ucam = 2.7%.

3. Overall uncertainty

The total uncertainty of the spectral-image fusion temperature reconstruction is as follows:

(22) $$u_{\mathrm{fusion}}=\sqrt{u_{\mathrm{spec}}+u_{\mathrm{cam}}+u_{\mathrm{map}}}$$

where $u_{\mathrm{map}}$= 0.8% is the uncertainty introduced by the grayscale-to-temperature-element mapping process. With $u_{\mathrm{spec}}$ = 2.1%, $u_{\mathrm{cam}}$ = 2.7%, and $u_{\mathrm{map}}$ = 0.8%, the combined relative standard uncertainty of the spectral-image fusion method is approximately 3.5%.

It should be noted that the relative differences between the proposed method and conventional spectral inversion at the four time points (3.2%, 3.4%, 3.3%, and 3.4%) reflect the consistency between the two methods under the current calibration conditions and are consistent with this calculated uncertainty bound. These values should not be interpreted as the overall absolute uncertainty of the measurement system.

## 8. Conclusions and Prospects

This study focused on the high-temperature transient characteristics of deflagration fireballs. A spectral-image fusion device was developed, and a method for reconstructing the two-dimensional temperature field by fusing synchronized spectral and imaging data was proposed. The measured spectra were calibrated, smoothed, normalized, and fitted using nonlinear least-squares spectral unmixing based on blackbody radiation theory. The optimized temperature-element weights were then combined with CCD grayscale images to reconstruct the two-dimensional temperature distribution of the deflagration fireball.

Experimental verification demonstrated a PRNU value below 2.2% at 9 m, confirming acceptable photo-response uniformity. Both the spectrometer and CCD sensor were operated at 340 fps with a 1 ms integration time, yielding a theoretical acquisition interval of approximately 2.94 ms. The four consecutive frames at 189, 192, 195, and 198 ms after ignition validated the millisecond-resolution spectral-image acquisition capability of the system.

The average temperatures reconstructed by the proposed method at these four time points were 1382, 1373, 1366, and 1357 K, while the corresponding conventional spectral inversion temperatures were 1430, 1422, 1414, and 1406 K, yielding relative errors of 3.2%, 3.4%, 3.3%, and 3.4% (average 3.3%). The combined relative standard uncertainty of the system was calculated to be approximately 3.5%. These results confirm that the proposed method achieves good consistency with conventional spectral inversion while additionally providing two-dimensional spatial temperature distribution. The reconstructed temperature maps show that the high-temperature region is concentrated near the fireball center, with temperature decreasing toward the edge.

Several limitations should be acknowledged. The wavelength-dependent emissivity of combustion products and the radiative transfer effects of incandescent gas and smoke were not independently measured; therefore, the reconstructed temperature represents an effective temperature under the graybody approximation. The maximum temperature observed in the experiment was approximately 2050 K at 6 ms, which reflects the current conditions rather than the system’s absolute measurement limit. Higher temperatures can be accessed by adjusting the integration time, neutral-density attenuation, and calibration range to avoid detector saturation. The reported relative errors and uncertainty (3.3–3.5%) are specific to the present hardware configuration; applying the method to different systems requires recalibration of the spectrometer response, CCD PRNU, geometric registration, exposure settings, and synchronization parameters.

Future work will focus on three aspects: (1) improving accuracy through emissivity correction, gas-transmission modeling, and high-dynamic-range imaging sensors; (2) enhancing temporal performance by optimizing the reconstruction algorithm toward real-time temperature field imaging; and (3) extending applicability through multi-angle imaging, controlled flame calibration experiments, and validation against independent reference measurements.

## Figures and Tables

**Table 1:** Main hardware specifications of the spectral-image fusion temperature measurement system.

Component	Model/Type	Main Parameters	Function
CCD camera	MV-CA003-21UM	0.3-megapixel 1/4″ CCD USB 3.0 industrial area-scan camera; sensor model: OnSemi PYTHON300; global shutter; monochrome; resolution: 640 × 480 pixels; pixel size: 4.8 μm × 4.8 μm; spectral response: 300–1100 nm; maximum frame rate: 814.5 fps at 640 × 480; experimental frame rate: 340 fps; dynamic range: 59 dB; signal-to-noise ratio: 39.9 dB; gain: 0–15 dB; exposure time: 40 μs–10 s	Acquisition of fireball images and grayscale distribution
Imaging lens	Industrial fixed-focus lens	Focal length: 100 mm; aperture diameter: 29 mm	Imaging the deflagration fireball onto the CCD sensor
Fiber-optic spectrometer	Ocean Optics USB2000+	Spectral range: 372.05–1047.04 nm; integration time: 1 ms; frame rate: 340 fps; spectral resolution: 0.3~10.0 nm	Acquisition of fireball radiation spectra
Fiber	Visible-near-infrared quartz fiber	Connector type: SMA 905; fiber core diameter: 400 μm; applicable wavelength range: 300–1100 nm	Transmission of fireball radiation to the spectrometer
Fiber collimator	FC-COL-1825	Collimator aperture: 18 mm; focal length: 25 mm	Collection and coupling of fireball radiation into the optical fiber
Standard blackbody furnace	DY-HT3	Temperature range: 300–1200 °C; maximum reachable temperature: 1200 °C; temperature resolution: 0.1 °C; emissivity: ≥0.995; power supply: 220 V AC, 50 Hz; stabilization time: 30 min at each temperature point	Calibration of the spectrometer response function

**Table 2:** Numerical validation results using simulated standard blackbody spectra.

Blackbody Set Temperature/K	Retrieved Temperature/K	Absolute Error/K	Relative Error/%
850	858	8	0.9
950	941	8	0.8
1050	1061	11	1.0
1150	1138	11	1.0

**Table 3:** Instrument parameter table.

Changed Hardware	Possible Changed Parameter	Influence on Measurement Error	Required Correction
CCD sensor	PRNU, dynamic range, noise level, spectral sensitivity	Affects grayscale-to-temperature mapping and spatial temperature reconstruction	PRNU calibration, dark-field/flat-field correction, exposure optimization
Fiber-optic spectrometer	Spectral response, wavelength accuracy, signal-to-noise ratio	Affects spectral correction and temperature-element fitting	Blackbody calibration and wavelength calibration
Lens or fiber collimator	Focal length, aperture, fiber core diameter, field of view	Affects collected radiation intensity and spatial correspondence	Geometric calibration and field-of-view matching
Optical-axis alignment	Relative position between CCD and spectrometer	Causes spatial registration error between image and spectrum	Optical alignment and image registration calibration
Acquisition system	Frame rate, integration time, trigger delay	Causes temporal mismatch for transient fireball evolution	Synchronization calibration

**Table 4:** Quantitative comparison between conventional spectral inversion and the proposed method.

Time/ms	Temperature Obtained by Conventional Spectral Inversion/K	Average Temperature Reconstructed by the Proposed Method/K	Absolute Error/K	Relative Error/%
189	1430	1382.	47	3.2
192	1422	1373	48	3.4
195	1414	1366	47	3.3
198	1406	1357	48	3.4
Average	1418	1370	47	3.3

**Table 5:** Comparison between the proposed method and alternative temperature measurement methods.

Method	Input Data	Temperature Output	Spatial Temperature Distribution	Main Limitation
Conventional spectral inversion	Corrected spectrum	Equivalent average temperature	No	Cannot directly reconstruct two-dimensional temperature distribution.
Imaging-only method	CCD/high-speed grayscale image	Grayscale or intensity distribution	Yes	Grayscale intensity cannot be directly converted into temperature without spectral constraints.
Tomographic reconstruction	Multi-view images or projections	Spatial temperature field	Yes	Requires multi-angle optical access, strict geometric calibration, and complex reconstruction algorithms; not applicable to the present single-view dataset.
Proposed spectral-image fusion method	Corrected spectrum + CCD grayscale image	Average temperature + two-dimensional temperature map	Yes	Accuracy is affected by spectral calibration, emissivity, gas transmission, image registration, and synchronization.

**Table 6:** Temperature measurement speed of the proposed method.

Item	Value
Integration time of fiber-optic spectrometer	1 ms
Integration time of CCD sensor	1 ms
Frame rate of fiber-optic spectrometer	340 fps
Frame rate of CCD sensor	340 fps
Theoretical acquisition interval	2.94 ms
Time interval of selected consecutive frames	3 ms

## Data Availability

Data supporting the results of the study can be accessed upon reasonable request from the corresponding author.
